# The Structure of Dipolar Polymer Brushes and Their Interaction in the Melt. Impact of Chain Stiffness

**DOI:** 10.3390/polym12122887

**Published:** 2020-12-02

**Authors:** Ivan V. Mikhailov, Victor M. Amoskov, Anatoly A. Darinskii, Tatiana M. Birshtein

**Affiliations:** Institute of Macromolecular Compounds, Russian Academy of Sciences, 199004 St. Petersburg, Russia; amoskovvm@yandex.ru (V.M.A.); a.darinskii@mail.ru (A.A.D.); tatiana.birshtein@gmail.com (T.M.B.)

**Keywords:** polymer brushes, interaction, dipoles, polymer melt

## Abstract

By using the numerical lattice Scheutjens–Fleer self-consistent field (SF-SCF) method we have studied the effect of the restricted flexibility of grafted chains on the structure and mutual interaction of two opposing planar conventional and A-type dipolar brushes. Brushes are immersed in the solvent consisting of chains similar to the grafted ones. The increase of the chain rigidity enhances the segregation of grafted chains in a A-type dipolar brush into two populations: backfolded chains with terminal monomers near the grafting surface and chains with the ends at the brush periphery. The fraction of backfolded chains grows by an increase of the Kuhn segment length. It is shown that two opposite A-type dipolar brushes from semi-rigid chains are attracted to each other at short distances. The attraction becomes more pronounced and begins at larger distances for more rigid chains with the same brush characteristics: polymerization degree, grafting density, and dipole moments of monomer units. This attraction is connected with the dipole-dipole interactions between chains of oncoming brushes with oppositely directed dipoles penetrating deeply into each other upon contact. This effect of the chain rigidity is opposite to that for conventional brushes without dipoles in the chains. For such brushes, an increase in the chain rigidity leads to the enhanced repulsion between them.

## 1. Introduction

Polymer brushes are layers of polymer chains with the end link firmly (almost irreversibly) connected to the surface. Recently, the anniversary of their appearance in polymer science was celebrated [1,2]. They are widely used for various modifications of the properties of surfaces [3,4,5]. One of the areas of application of polymer brushes is a stabilization of colloidal and nanoparticles in solutions and filler nanoparticles in polymer nanocomposites. First of all, decorating of the particle by the brush has to provide a possibility to include particles into the matrix, even by the mutual incompatibility of them [3,4,5,6]. On the other hand, the distribution of such particles in the medium depends on the interaction between grafted brushes. If the brushes repel each other, this helps to avoids segregation of polymer modified particles and ensures a more uniform distribution of them in the matrix. The structure of the brush and its interaction with other brushes depends on the molecular characteristics of grafted chains such as the chemical structure and the molecular weight, charging, the grafting density, the matrix properties as well as on the geometry of the surface. In the present work, we will limit ourselves to considering planar polymer brushes formed by neutral homopolymers.

There are a lot of works where so-called conventional brushes were studied theoretically. The structure of such brushes depends on the solvent strength and is determined by a compromise between the tendency to decrease the brush density due to volume interactions and the need for this to stretch the grafted chains, losing conformational entropy. In a good or θ-solvent (Flory–Huggins parameter χ ≤ 0.5) the brush is “wet”, it contains a large amount of solvent, becoming “dry” only at extremely high grafting density. The main feature of the structure is the strong extension of the grafted chains and a wide distribution in the degree of extension. The brush is not uniform in the density, which is maximum at the grafting surface and drops to zero towards the periphery (see, for example, the review [7]). The opposing brushes are virtually mutually impenetrable and repel each other when approaching due to the volume interactions.

The substitution of the low molecular solvent by the high molecular melt changes both the structure of conventional brushes and the interaction between them. Such a situation, in particular, arises in nanocomposites when nanofillers decorated by the brush are immersed into the polymer melt consisting of flexible chains chemically identical to the grafted ones. The chemical identity of the grafted chains in the brush and the macromolecules of the polymer melt (χ = 0) is a sufficient (although not necessary) condition to overcome incompatibility between the filler and the melt. Under these conditions, free polymer chains of the melt screen the volume interactions of the monomer units of grafted macromolecules. As a result, the brush becomes denser than in the solution, and the stretching degree of grafted chains decreases. With sparse grafting, the chains do not stretch, so the brush is a system of overlapping Gaussian coils and contains free polymer (“wet” brush). At large grafting densities, the stretched chains form an almost dry brush without melt chains (except for the peripheral part) [2,8,9].

The theoretical study of the problem of the mutual interaction of such brushes in a polymer matrix has a long history [8,9,10,11,12,13,14]. The main result is the existence of the weak short-range attraction between them. This attraction is connected with the tendency of grafted chains of the brush to mix more easily with grafted chains from the other brush than with the free polymer chains in the melt. The above-mentioned conclusions concerning the structure and interaction of conventional brushes in different media were confirmed in a large number of experimental and computer simulation studies (see [7] for a review).

Recently, Glova et al. [15,16] have found a new “unusual structure” of a brush in a chemically equivalent polymer environment. By using the fully atomistic molecular dynamics (MD), they have studied the structure of the brush consisting of the lactic acid oligomers (OLA) covalently grafted to the cellulose nanocrystals (CNC) immersed into the polylactic acid (PLA) matrix. Polylactide-based bionanocomposites are a promising class of materials [12,17]. It turned out that the grafted chains separate in two populations: the extended chains with the ends on the brush periphery and backfolded ones («hairpins») with the ends near the grafting surface. The analysis has shown that such an unusual behavior is caused by the interaction of atomic partial charges in the brush. By exclusion of electrostatic interactions, the brush structure becomes similar to that for conventional brushes.

Theoretical studies [18] made by using the lattice Scheutjens–Fleer self-consistent field (SF-SCF) method have shown that such an unusual structure arises due to the presence of longitudinal dipole moments in grafted macromolecules. These dipole moments are connected with the distribution of partial charges and are directed along the monomers of the grafted chains. When all chains of the brush are grafted onto the surface via the same end, the longitudinal dipoles of all stretched chains are directed in the same way. This causes the appearance of the population of backfolded chains.

Note that the longitudinal component of the dipole moment can arise in macromolecules with three or more different bonds in monomer units of the main chain (polyesters, in particular PLA, or polypeptides, for example). For reasons of symmetry polar macromolecules with two different bonds in the monomer unit such as, for example, polyvinyl chloride, cannot have a longitudinal component of the dipole moment [19]. Following the classification of Stockmayer [17], in what follows, we will call macromolecules with longitudinal dipole moments macromolecules of type A. We will also call the brushes formed by A-type chains, which are grafted with the same ends A-type brushes.

The obvious question arises how this unusual structure will affect the mutual interactions of brushes in polymer medium. The answer to this question is one of the goals of the present work. It should be mentioned the theoretical works where dipolar brushes in polar solvent [20,21,22,23] were considered. In contrast to our case the directions of dipoles in the grafted chains were not correlated with the directions of monomer units. The dipole moments of macromolecules in this case do not contain longitudinal components and are directed perpendicular to the chain length vectors (contain only a transverse components). Such macromolecules and brushes made of such grafted macromolecules are not type A systems, which are considered in our work. Kumar et al. [20], Mahalik et al. [21], Budkov et al. [22], Gordievskaya et al. [23], show that the effect of dipole-dipole interaction between transverse dipoles can be described by the introduction of the macroscopic effective Flory–Huggins-like parameter which depends on the polymer concentration. These interactions can lead to the collapse of the dipolar flat brushes and to an attraction between two opposite brushes at intermediate separation distances [24]. To avoid misunderstandings, A-type brushes were not considered in these works.

Another goal of the present work is to study the influence of the limited flexibility of the grafted chains and free chains in the polymer matrix on the internal structure and interaction of brushes. The formulation of this problem is motivated by the results of further atomistic MD modeling [25], which demonstrated the difference in the structure of brushes formed on the surface of CNCs particles by grafted OLA and oligohydroxybutyrate (OHB) in melts of PLA and polyhydroxybutyrate (PHB) chains, respectively. The fraction of “hairpins” in the OLA brush was larger than that in the OHB brush at the same grafting densities. The authors have concluded that this difference is caused by the lower flexibility of PLA macromolecules as compared to PHB ones.

It should be noted that the effect of limited flexibility of macromolecules on the structure and mutual interaction of brushes in the polymer melt has not been studied even for conventional brushes. For conventional brushes in solution, such an analysis was carried out already at the earliest stage in the development of theoretical studies of brushes [10,13,14].

All the studies in the present work were performed by using the numerical lattice SF-SCF method, which has proven effective for many polymer systems, including polymer brushes.

The outline of the paper is the following one. In Section 2, the model of the brush and method are described. The diagram of states of a brush formed by macromolecules of limited flexibility in a polymer melt and the results for a single planar dipolar brush immersed into the melt will be presented in Section 3.1. The effect of such parameters as the polymerization degree *N*, grafting density *σ*, parameter τ of the dipole-dipole interactions, and the Kuhn segment length *p* on the brush structure will be studied. In Section 3.2, the effect of the above-mentioned parameters on the mutual interaction of two such brushes will be studied. Section 4 concludes the paper.

## 2. Model and Method

In present work, a coarse-grained model of the planar polymer brushes consisting of polymer chains pinned by one end to a flat impenetrable surface was used. It is assumed that all monomer units in chain are identical and have the same linear size *a*. The value of *a* is used as the unit length.

The grafting density of the chains is characterized by the dimensionless ratio *σ* = *a*^2^/*s*, where *s* is an average surface area per one grafted macromolecule.

To investigate the properties of dipolar brushes, the Scheutjens–Fleer self-consistent field (SF-SCF) method [26,27] was used. In the SF-SCF method, all the various interactions between the particles of the system are taken into account not explicitly but through a mean effective field acting on the given particle. This reduces the problem of the determination of properties of the system consisting of many polymer chains to the problem of the behavior of a single chain in the effective field. This problem is quite easy to solve with a relatively low computational cost. The disadvantage of the mean field approximation as compared with more detailed methods (Monte-Carlo, molecular or stochastic dynamics) is its inability to take into account the correlations in the system. However, this disadvantage weakly affects the statistical properties of the brushes. Advantages of SF-SCF method are high computational efficiency combined with the accuracy of the results reproducibility.

We use the lattice numerical SF-SCF method. The space is taken to be composed of cubic lattice sites with characteristic length a and the volume per cell *a*^3^. Due to the symmetry of the system all brush characteristics change only along z axis directed normally to the grafting surface. The space is divided into layers parallel to the surface which are numbered from *z* = 0 to *z_max_*. Layer *z* = 0 corresponds to the grafting surface, the first segments of all grafted chains are located in the layer *z* = 1. When two opposite brushes are considered, the opposite grafting surface is located in the layer (*z* = *z_max_* + 1) and the first segments of chains belonging to another brush are in the layer *z* = *z_max_*. The distance between the two grafting surfaces is *D* = *z_max_*·*a*. The chain grafting is supposed to be sufficiently dense, so the chains overlap strongly and form a relatively uniform brush. Under these conditions, the polymer volume fraction *ϕ* and the exchange chemical potential *u* remain constant within the same layer and have one nonzero gradient in *z*-direction.

The grafted chains are modeled as lattice chains with restricted flexibility in an external effective field *u*(*z*), which describes the effect of the inter- and intramolecular interactions. Two adjacent monomer units of the chain occupy neighboring lattice sites. However, the long range correlations are ignored. Hence, the overlap of two monomer units on the same lattice site is, in principle, allowed, but is prevented effectively through the incompressibility condition, which is to be fulfilled in each layer:

(1) $$\underset{X}{\sum }\varphi_{X}\left(z\right)=1,\;X=1,2,s.$$

Hereinafter, the subscripts indicate that the parameter belongs to the polymer chain of the type 1, type 2 or to the solvent (*s*) molecule, respectively.

The chain stiffness is characterized by the Kuhn segment length *p*, which obeys the equation [28]:

(2) $$\frac{p}{a}=\frac{1+\langle \cos\gamma \rangle }{1-\langle \cos\gamma \rangle }$$

where *γ* = *π* − *θ* is the supplementary angle to the valence angle *θ*.

In this model, the correlation between two consecutive chain links is given by the potential:

(3) $$\frac{U_{a}\left(\gamma \right)}{k_{B}T}=K\left(1-\cos\gamma \right),$$

where *K* is a coefficient of the bonds correlation.

On the cubic lattice, two consecutive bonds can have three relative orientations: (1) straight conformation, where a bond makes an angle *θ* = *π* with the preceding one (that corresponds to *γ* = 0 and *U_a_*^(*s*)^ = 0); (2) perpendicular kink (*γ* = *π*/2 and *U_a_*^(*p*)^ = *K*)); (3) backfold “fracture” (*γ* = *π* and *U_a_*^(*b*)^ = 2*K*). The weighting factor *λ_s_* of the straight conformation and the weighting factor *λ_b_* of the backfold conformation are related to the weighting factor *λ_p_* of the perpendicular kink as

(4) $$\lambda_{s}=\lambda_{p}\exp\left(-\frac{U_{a}^{\left(s\right)}}{k_{B}T}\right)/\exp\left(-\frac{U_{a}^{\left(p\right)}}{k_{B}T}\right)=\lambda_{p}\exp K$$

(5) $$\lambda_{b}=\lambda_{p}\exp\left(-\frac{U_{a}^{\left(b\right)}}{k_{B}T}\right)/\exp\left(-\frac{U_{a}^{\left(p\right)}}{k_{B}T}\right)=\lambda_{p}\exp\left(-K\right)$$

The sum of the weighting factors in the case of cubic lattice obeys the normalization condition:

(6) $$\lambda_{s}+4\lambda_{p}+\lambda_{b}=1$$

The average cosine of the angle *γ* can be calculated by the equation:

(7) $$\langle \cos\gamma \rangle =\lambda_{s}\cos0+4\lambda_{p}\cos\frac{\pi }{2}+\lambda_{b}\cos\pi =\lambda_{s}-\lambda_{b}$$

According to the above Equations (2) and (4)–(7), weight coefficient *λ_p_* can be found by the formulae:

(8) $$\lambda_{p}={\left(\exp K+\frac{1}{\exp K}+4\right)}^{-1},$$

where

(9) $$\exp K=\sqrt{{\left(1-\frac{p}{a}\right)}^{2}+\frac{p}{a}}+\frac{p}{a}-1$$

Thus, using expressions (4), (5), (8) and (9), the weighting factors (*λ_b_*, *λ_p_*, *λ_s_*) for different orientations of adjacent segments along the chain are uniquely determined through the length Kuhn segment, *p*. Note that the given model does not prohibit the reverse movement along the chain and allows the overlap of segments, but such overlapping is prevented through the incompressibility condition.

The grafted chains are immersed in the melt of exactly the same chains. The free energy per unit area of the grafting surface is calculated as the energy of dipole-dipole interactions *F_d−d_* and the sum of the negative logarithms of the partition functions *Q_X_* of all components (*X* = 1,2,*s*) in a constant effective external fields $u_{X}\left(z\right)$ minus the work of these fields $\sum_{X}u_{X}\left(z\right)\varphi_{X}\left(z\right),$ (according to the Legendre transform):

(10) $$F\left\{u,\varphi \right\}=F_{d-d}\left\{\varphi_{X}\left(z\right)\right\}-k_{B}T\underset{X,X\neq s}{\sum }\sigma_{X}\ln Q_{X}\left\{u_{X}\left(z\right)\right\}-\underset{z}{\sum }\underset{X}{\sum }u_{X}\left(z\right)\varphi_{X}\left(z\right),$$

where *σ_X_* is the *X*-chain grafting density.

The main goal of SCF calculations is to determine the system characteristics which correspond to the minimum of the free energy under the incompressibility condition. This goal is achieved through the optimization of the functional:

(11) $$ℱ=F\left\{u,\varphi \right\}+\underset{z}{\sum }\alpha \left(z\right)\left[\underset{X}{\sum }\varphi_{X}\left(z\right)-1\right],$$

where *α*(*z*) is Lagrange field (set of Lagrange multipliers). The Lagrange field *α*(*z*) corresponding to the minimum of functional ℱ (Equation (11)) is calculated during the iterative procedure of gradient descent:

(12) $$\alpha \left(z\right)\leftarrow \alpha \left(z\right)+\eta \left[\underset{X}{\sum }\varphi_{X}\left(z\right)-1\right],$$

where *η* < 1/2 is the convergence step size, which corresponds to the highest convergence rate.

Minimization of ℱ with respect to the volume fractions *ϕ_X_*(*z*) allows to calculate the potential fields *u_X_*(*z*):

(13) $$\frac{\delta ℱ}{\delta \varphi_{X}\left(z\right)}=0\Rightarrow u_{X}\left(z\right)\leftarrow \alpha \left(z\right)+\frac{\delta F_{d-d}\left\{\varphi_{X}\left(z\right)\right\}}{\delta \varphi_{X}\left(z\right)}$$

Minimization of ℱ with respect to the potential fields *u_X_*(*z*) provides a way to calculate the volume fraction distributions *ϕ_X_*(*z*):

(14) $$\frac{\delta ℱ}{\delta u_{X}\left(z\right)}=0\Rightarrow \varphi_{X}\left(z\right)\leftarrow \sigma_{X}\frac{\delta \left(-\ln Q_{X}\right)}{\delta u_{X}\left(z\right)}$$

For the calculation of the energy of dipole-dipole interactions *F_d−d_* we apply a mean-field approach used in [18]:

(15) $$\frac{F_{d-d}}{k_{B}T}=\frac{\tau }{2}\underset{z}{\sum }{\left(\underset{X}{\sum }\varphi_{X}\left(z\right)s_{1,X}\left(z\right)\right)}^{2}$$

Here *τ* is the dimensionless parameter of dipole-dipole interactions (which is proportional to the dipole moment µ of the monomer unit), *s*_1_(*z*) is the first order parameter of dipoles in *z*-th layer. Based on the assumption that the dipoles are directed along the bonds, the order parameter has the form:

(16) $$s_{1}\left(z\right)=\frac{\varphi \left(z,d=1\right)-\varphi \left(z,d=-1\right)}{\varphi \left(z,d=-1\right)+\varphi \left(z,d=0\right)+\varphi \left(z,d=1\right)}\;=\frac{\varphi \left(z,d=1\right)-\varphi \left(z,d=-1\right)}{\varphi \left(z\right)},$$

where *ϕ*(*z*, *d* = 1) and *ϕ*(*z*, *d* = −1) are volume fractions of segments oriented in different directions *d* relative to the grafting surface. There are three possible directions d of monomer unit in each lattice layer: from layer *z* to layer *z* − 1 (*d* = −1), within layer *z* (*d* = 0), or from layer *z* to layer *z* + 1 (*d* = 1). The potential of dipole-dipole interactions acts on the monomer depending on its orientation in space:

(17) $$u_{d-d}\left(z,d\right)=\frac{\delta F_{d-d}\left\{\varphi \left(z,d\right)\right\}}{\delta \varphi \left(z,d\right)}=\frac{d}{\left|d\right|}\tau \varphi \left(z\right)s_{1}\left(z\right)$$

The value of *τ* can be expressed as:

(18) $$\tau \sim \frac{\mu^{2}}{\pi \varepsilon_{0}\epsilon_{r}a^{3}k_{B}T},$$

where $\epsilon_{r}$ is the relative dielectric constant of the polymer melt and $\varepsilon_{0}$ is the vacuum electric permittivity.

The analytical solution of Equations (13) and (14) for the case of polymer molecules is rather difficult. They are solved by using the iterative procedure which also takes into account the linking of monomer units in chains.

The relative preference of any monomer unit to be in layer z with respect to the bulk melt is determined by the statistical Boltzmann weight:

(19) $$W_{B}\left(z,d\right)=\exp\left(-\frac{u\left(z,d\right)}{k_{B}T}\right)$$

The chain conformation is considered as a set of trajectories of particle diffusion from fixed to free end (forward propagate) and vice versa from free to fixed end (back propagate). Both processes are characterized by the probability density matrices of monomer units. These matrices are called forward *G_f_* and back *G_b_* propagators. Propagation matrices also differ in directions (*d* = −1, 0, 1). The way to “fill” these matrices is as follows: first, initial conditions are set up. For instance, for a polymer grafted to a surface (*z* = 0), the initial conditions are as follows

(20) $$G_{f}\left(z=0,s=0,d=1\right)=W_{B}\left(z=1,d=1\right),\;\;G_{b}\left(z,s=N,d\right)=W_{B}\left(z,-d\right),$$

which means that the first monomer unit (*s* = 1) of the chain is fixed in layer *z* = 1, and the last segment (*s* = *N*) can be in all accessible layers. In the case of a solvent molecule, which is also a chain of length *N*, both end segments are free:

(21) $$G_{f}\left(z,s=1,d\right)=W_{B}\left(z,d\right),\;\;G_{b}\left(z,s=N,d\right)=W_{B}\left(z,-d\right),$$

Each next step of the propagators *G_f_*(*z*,*s*,*d*) and *G_b_*(*z*,*s*,*d*) is computed during iterations respect with *s* and *z*. This procedure is described in detail in [29,30].

The statistical weight *q*(*z*,*s*,*d*) of the chain conformations in which the *s*-th segment with orientation *d* is in the *z*-th layer can be represented as a composition of two propagators (the composition law) [27]:

(22) $$q\left(z,s,d\right)=\frac{G_{f}\left(z,s,d\right)\cdot G_{b}\left(z,s,-d\right)}{W_{B}\left(z,d\right)}$$

The total partition function of chain is equal to

(23) $$Q=\overset{N}{\underset{s=1}{\sum }}\overset{z_{max}}{\underset{z=1}{\sum }}\left[q\left(z,s,-1\right)+4q\left(z,s,0\right)+q\left(z,s,1\right)\right]$$

The product of propagators gives the volume fraction profile:

(24) $$\varphi \left(z\right)=C\overset{N}{\underset{s=1}{\sum }}\left[\frac{G_{f}\left(z,s,-1\right)\cdot G_{b}\left(z,s,1\right)}{W_{B}\left(z,-1\right)}+4\frac{G_{f}\left(z,s,0\right)\cdot G_{b}\left(z,s,0\right)}{W_{B}\left(z,0\right)}+\frac{G_{f}\left(z,s,1\right)\cdot G_{b}\left(z,s,-1\right)}{W_{B}\left(z,1\right)}\right],$$

where *C* is a normalization constant. For the free chain in melt *C* = 1/*N*. For the polymer grafted in the first layer, this constant is equal to

(25) $$C=\sigma {\left[G_{b}\left(1,1,-1\right)+4G_{b}\left(1,1,0\right)+G_{b}\left(1,1,1\right)\right]}^{-1}$$

that ensures the normalization of the volume fraction profile:

(26) $$\underset{z}{\sum }\varphi \left(z\right)=N\sigma$$

The volume fraction profile can also be split into three components depending on the segment orientation:

(27) $$\varphi \left(z,d\right)=C\overset{N}{\underset{s=1}{\sum }}\left(4-3\left|d\right|\right)\frac{G_{f}\left(z,s,d\right)\cdot G_{b}\left(z,s,-d\right)}{W_{B}\left(z,d\right)}$$

Under described approach, chain conformations are considered using a second-order Markov model. This implementation of Markov formalism for chain stiffness was proposed in pioneering works [29,30,31,32].

To undertake the above calculations, the special software was developed.

## 3. Results and Discussion

### 3.1. Structure of Single Polymer Brushes in the Melt

In this section, we will consider the structures of polymer brushes in the melt with the similarity of grafted chains in the brush and free chains in melt (*χ* = 0). The goal is to determine the effect of two factors on the brush structure: the limited flexibility of chains (*p* > 1) and the dipole-dipole interactions of longitudinal dipoles in A-type brushes (*τ* > 0).

#### 3.1.1. Density Profiles and State Diagram of Brushes in the Melt

**Figure 1:**
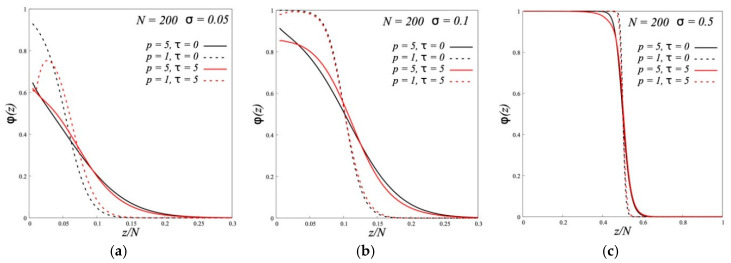
The volume fraction profiles *ϕ*(*z*) of polymer brushes with different stiffness of the grafted chains (*p*) and different values of the parameter of dipole-dipole interactions (*τ*) in melt of the same chains at grafting densities *σ* = 0.05 (**a**), *σ* = 0.1 (**b**) and *σ* = 0.5 (**c**).

Figure 1 show, as typical examples, the volume fraction profiles of polymer brushes calculated by the SF-SCF method at very low (*σ* = 0.05), moderately low (*σ* = 0.1), and high grafting densities (*σ* = 0.5). For each value of σ, the volume fraction profiles for four different brushes are compared, including two dipolar brushes of type A (*τ* = 5) and two conventional nondipolar brushes (*τ* = 0), that differ in the flexibility of the chains (*p* = 1 and *p* = 5). The polymerization degree of grafted chains in the brush and free chains in the melt is *N* = 200, the chains in the melt are characterized by the same values of *τ* and *p* as the brush chains.

As can be seen from Figure 1, the main effect on the brush density profiles is provided by the flexibility of grafted chains. For all values of *σ*, the curves for *p* = 1 and *p* = 5 are very different, but only slightly differing and even coinciding for conventional and for dipolar brushes of type A. At very sparse grafting (*σ* = 0.05), the average density of brushes, both from flexible (*p* = 1) and semi-rigid (*p* = 5) chains, is small, and the brushes contain a large amount of free polymer in the entire volume. Reducing the chain flexibility leads to the loosening of the brush and the brush thickness increases. With a high grafting density (*σ* = 0.5), brushes made of both flexible and semi-rigid chains have a maximum density of *ϕ* = 1 in the inner part. Reduced flexibility leads to a gentler drop in brush density from *ϕ* = 1 to *ϕ* = 0 at the brush periphery. This periphery, unlike the inner part of the brushes, contains a free polymer. The strongest influence of flexibility on the volume fraction profiles is observed in Figure 1 at *σ* = 0.1. The structure of a brush made of flexible chains under these conditions is similar to the structure at large *σ*, and the structure of a brush made of semi-rigid chains is similar to the structure at small *σ*.

Figure 1 allows to conclude that for brushes made of semi-rigid chains, as for brushes made of flexible chains, two regimes are possible. The position of the boundary σ^*^ between these regimes depends on the flexibility of the grafted chains. We checked this conclusion for the conventional brushes, by generalization of a scaling diagram of states for a brush of flexible chains (*p* = 1) of length *N* in a melt of chains of length *M* previously constructed in [11], for the case of semi-rigid chains *p* >1. The derivation of this diagram is given in the Appendix A.

White area in the diagram corresponds to the regimes where the grafted chains are stretched beyond their equilibrium Gaussian dimensions (*H* > (*N p*)^1/2^). In this area one can distinguish three regimes with different power law dependences for interpenetration length Δ, corresponding to full (I), partial (II), and peripheral (III) penetration of the free chains of the melt into the brush. In the green area of the diagram (regime IV) the polymer chains in the brush are not stretched and their end-to-end distance *H* is given by the equation (*H* = (*N p*)^1/2^) In this regime, the penetration length Δ is equal to the brush thickness (see Table 1).

**Figure 2:**
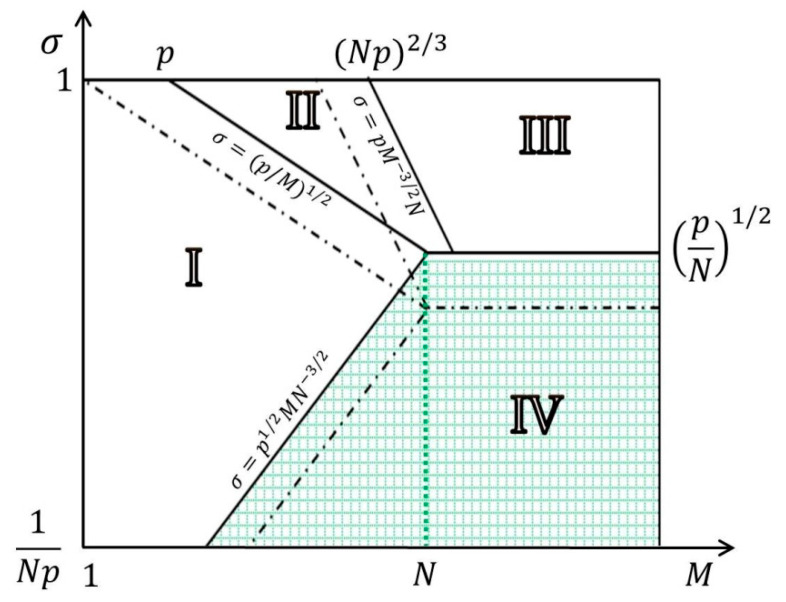
Diagram of states of a planar brush consisting of linear polymer chains with degree of polymerization *N* >> 1 immersed into melt of chemically identical linear polymer chains with degree of polymerization *M*. Green area corresponds to loss of the chain stretching, *H* ∼ (*N p*)^1/2^*a*. Regimes I, II, and III in the diagram correspond to full, partial, and peripheral penetration of solvent molecules into the brush, respectively. The boundaries dividing these regimes are indicated by dashed lines in the case of flexible chains (*p* = 1) and solid lines in the case of rigid chains (*p* > 1). The graph is plotted in log-log (*σ*, *M*) coordinates.

The state of the brush is characterized by a scaling dependence on the brush parameters of two brush characteristics: its thickness *H* and the depth Δ of penetration of free chains into the brush (the width of the region *ϕ* < 1). The comparison with the diagram for a brush of flexible chains (dashed line in Figure 2) shows that the diagram for a brush with semi-rigid chains remains the same, but with the shifted boundaries between regimes. The formulae for *H* and Δ, contain the dependencies on *p* (see Table 1). In the case of *M* = *N* (and for *M* > *N*), two regimes are possible: the regime IV of a “wet” brush made of overlapping Gaussian chains and the regime III corresponding to the “dry” brush containing the melt macromolecules only on the periphery. When the flexibility of grafted chains decreases, the boundary between the regimes σ^*^ = (*p/N*)^1/2^ shifts towards large values, and the region IV expands. The scaling formula for the boundary between regimes at *N* = 200 at *p* = 1 and at *p* = 5 gives for *τ* = 0 and *τ* = 5 the values of σ^*^ ≈ 0.07 and σ^*^ ≈ 0.15, respectively.

The results shown in Figure 1 are in agreement with these values (even without the introduction of a numerical factor). At *σ* = 0.05 both *p* = 1 and *p* = 5 correspond to the “wet” regime. At *σ* = 0.1 the brush of chains with *p* = 1 becomes “dry”, and the brush of chains with *p* = 5 remains “wet”. With *σ* = 0.5 both brushes are dry. Further, according to the formulae in Table 1 with decreasing flexibility (increasing *p*), the thickness of the “wet” brush in regime IV and the thickness of the density drop region in “dry” brushes in regime III should increase.

**Figure 3:**
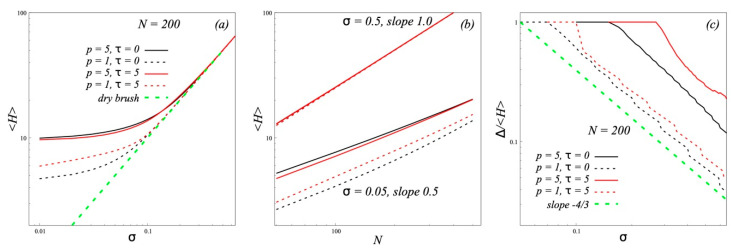
The thickness <*H*> of brushes with different stiffness of the grafted chains (*p*) and different values of the parameter of dipole-dipole interaction (*τ*) in melt of the same chains. The values <*H*> are plotted vs the grafting density *σ* (**a**) and the degree of polymerization N of the grafted chains (**b**). The graph (**c**) shows the ratio of the overlap zone Δ of free chains with the brush chains to the brush thickness <*H*>. All graphs are plotted in log-log coordinates.

Data, shown in Figure 3a,b, confirm the theoretical predictions for the dependence of the brush thickness *H* on *σ* at constant *N* (a) and on *N* at large and small *σ* (b).

The values of H were determined as the first moments <*H*> = (∑*_z_φ*(*z*)*z*)/(∑*_z_φ*(*z*)) of the density profiles *φ*(*z*) calculated by SF-SCF method. It is seen that at small σ (the regime IV) thickness *H* doesn’t depend on σ but its values are larger for semi-flexible chains *p* = 5. At large *σ* (the regime III) all curves *H*(*σ*) merge into one curve with the scaling slope equal to unity. The transition between regimes shifts to larger *σ* by an increase of *p*. The slope of dependencies *H*(*N*) changes from 0.5 at small *σ* to 1.0 at large *σ*.

The width of the overlapping zone Δ of grafted and free chains is calculated by SF-SCF lattice method by using the formula:

(28) $$\Delta =\frac{\sum_{z_{0}}^{z_{max}}\varphi \left(z\right)\left(z-z_{0}\right)}{\sum_{z_{0}}^{z_{max}}\varphi \left(z\right)},\;\;\varphi \left(z>z_{0}\right)<1$$

The ratio of Δ to the brush thickness <*H*> (Figure 3c) also shows agreement with the scaling theory. The overlapping zone of free chains begins to decrease at lower grafting densities in the case of more flexible chains. It should be noted that under the same conditions the value Δ/<*H*> is higher for dipolar brushes (*τ* = 5) relative to uncharged brushes (*τ* = 0) with the same chain flexibility. This effect can be explained by the additional penetration of free dipole chains into the brush to compensate longitudinal dipole moments of the grafted chains.

#### 3.1.2. Internal Structure

**Figure 4:**
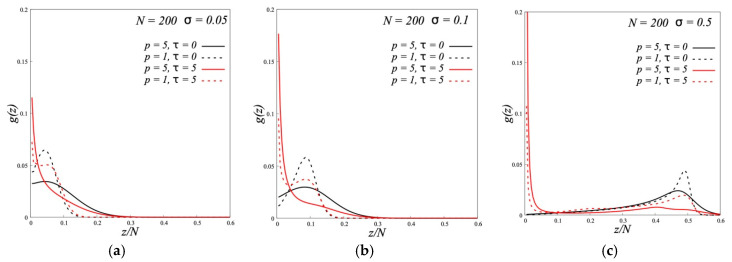
The end segment distributions *g*(*z*) in polymer brushes with different stiffness of the grafted chains (*p*) and different values of the parameter of dipole-dipole interactions (*τ*) in melt of the same chains at grafting densities *σ* = 0.05 (**a**), *σ* = 0.1 (**b**) and *σ* = 0.5 (**c**).

As we have shown above the effect of the dipole-dipole interactions on the density profiles of brushes is small. But if we look at the internal structure, we will see its drastic change when the dipoles were included. In conventional brushes the distribution of terminal monomer units shows one maximum at *z* > 1 which shifts to the brush periphery with the growth of σ (Figure 4). It means that the grafted chains are directed from the grafting surface to the brush periphery. An increase in Kuhn segment length *p* leads to the decrease and broadening of this maximum but the direction of the chains does not change. Such an effect of *p* on the ends distribution correlates with that for the density profiles (Figure 1). In dipolar brushes one observes the emergence of the second maximum near the grafting surface. This maximum corresponds to backfolded chains (“hairpins”). In the folded conformation the dipole moments of opposing parts of the chain have different signs and this conformation is favorable energetically (but unfavorable from the point of view of conformational entropy). Such unusual structure of dipolar brush with separation of chains into two groups was first demonstrated by MD modeling in [15,16,25] and theoretically investigated by SF-SCF method for A-type brush with flexible chains (*p* = 1) in [18].

**Figure 5:**
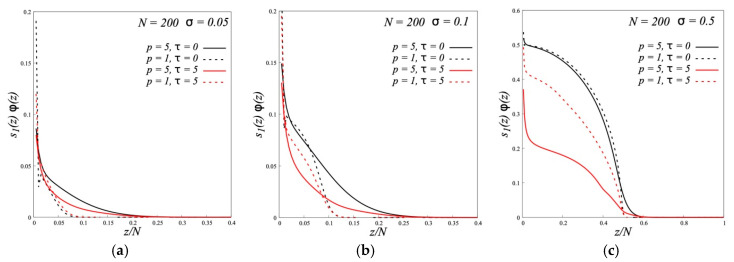
The segment orientation profile *s*_1_(*z*)*ϕ*(*z*) = *ϕ*(*z*, +1) − *ϕ*(*z*, −1) in polymer brushes with different stiffness of the grafted chains (*p*) and different values of the parameter of dipole-dipole interactions (*τ*) at grafting densities *σ* = 0.05 (**a**), *σ* = 0.1 (**b**) and *σ* = 0.5 (**c**).

**Figure 6:**
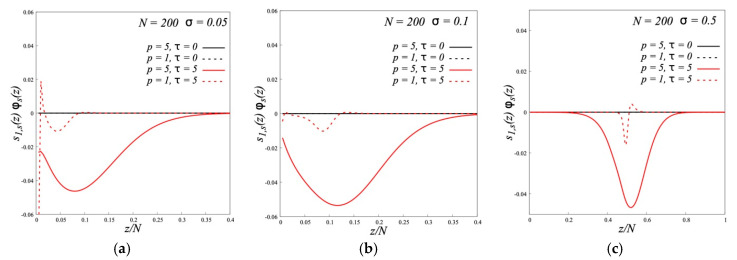
The segment orientation profile *s*_1,*s*_(*z*)*ϕ*(*z*) = *ϕ_s_*(*z*, +1) − *ϕ_s_*(*z*, −1) of free polymer chains in melt with different stiffness (*p*) and different values of the parameter of dipole-dipole interactions (*τ*) at grafting densities *σ* = 0.05 (**a**), *σ* = 0.1 (**b**) and *σ* = 0.5 (**c**).

The appearance of backfolded chains manifests itself in the *z*-dependence of the parameter of the segment orientation which is defined by the Equation (2). Figure 5 shows it for different *σ*. For all curves, the segments of grafted chains are oriented on average normal to the grafting surface and their orientation decreases by moving from the grafting point to the chain end. However, at same *p,* the curves for dipolar brushes are located below those for nonpolar brushes. The partial backfolding of chains in dipolar brushes leads to the decrease of *s*_1_ because the segments of neighboring chains in “hairpins” have alternative orientations. This effect is more pronounced in the dense brushes and increases with decreasing flexibility of the chains (growth *p*). For the solvent chains, which penetrate into the brushes, we see that they are oriented alternatively to the orientation of grafted chains (Figure 6). For semi-rigid chains this orientation is more pronounced and observed in the broader region than for the flexible chains.

Note that we consider the systems without any specific interactions between polymer chains and the grafting surface. It is the dipole-dipole interaction of the grafted chains in the A type brush that leads to the enrichment of the surface with free ends (Figure 4), as would be the case if they were attracted to the surface. When all segments of chains are attracted to the surface a different behavior was observed. Such a system, for example, was considered in [33], where silica particles with grafted PMMA chains in the PMMA melt were simulated by using full atomic MD. In this case, the segments of grafted chains located close to the surface are oriented along it, but more distant segments came to be oriented perpendicular to the surface. Their orientation increases as they approach the end. At same p the curves for dipolar brushes are located below those for nonpolar brushes.

The fraction of “hairpins” is determined by the balance between energy gain and entropy loss. Increase of the dipole-dipole interaction strength *τ* leads to the increase of the energetic gain by chain folding and an increase of the Kuhn segment length *p* leads to the decrease of the conformation entropy loss.

**Figure 7:**
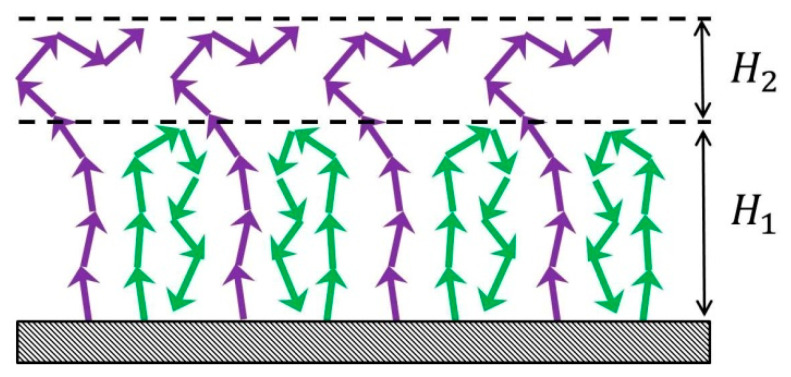
Schematic representation of the planar brush with two populations of chains (backfolded—green arrows and extended—violet arrows).

The above results for the density profile of the brush in the own melt demonstrate that for the dense grafting the effect of the chain rigidity is weak. Correspondingly the penetration of the high molecular solvent into the brush is small and the brush is practically “dry”. Therefore, for the semi-quantitative description of the effect of *p* on the dipolar brush structure, we can generalize the simple analytical theory developed in ref. [18] for the “dry” brush consisting of flexible (*p* = 1) dipolar chains. The theory is based on the two-state box model where all grafted chains are divided into two fractions: backfolded chains with terminal monomer units on the grafting surface and middle monomer units on the same hight and extended chains passing through the layer of the backfolded chains and finishing on the outer boundary of the brush (Figure 7).

The characteristics of the brush structure are fraction of folded chains *α* and fraction *β* of monomers of extended chains located in the inner layer. The free energy per chain consists of two parts: the energy of the deformation of chains in both layers *F_def_* and the energy of dipole-dipole interactions *F_int_*:

(29) $$F=F_{def}+F_{int}$$

A detailed description of the output of equations for *F_def_* and *F_int_* one can see in Appendix to the ref. [18]. The equilibrium values of *α* and *β* are determined by minimization of the free energy with respect to *α* and *β*. It is easy to show that the form of the dipole-dipole interaction does not change with *p*:

(30) $$\frac{F_{int}}{k_{B}T}=\frac{\tau }{2}\left(1-\alpha \right)N\sigma^{2}$$

and the deformation term is reduced by a factor of *p*:

(31) $$\frac{F_{def}}{k_{B}T}=\frac{3aN}{2p}\sigma^{2}\left\{{\left[\alpha +\left(1-\alpha \right)\beta \right]}^{2}\left(4\alpha +\frac{1-\alpha }{\beta }\right)+{\left(1-\alpha \right)}^{3}\left(1-\beta \right)\right\}$$

**Figure 8:**
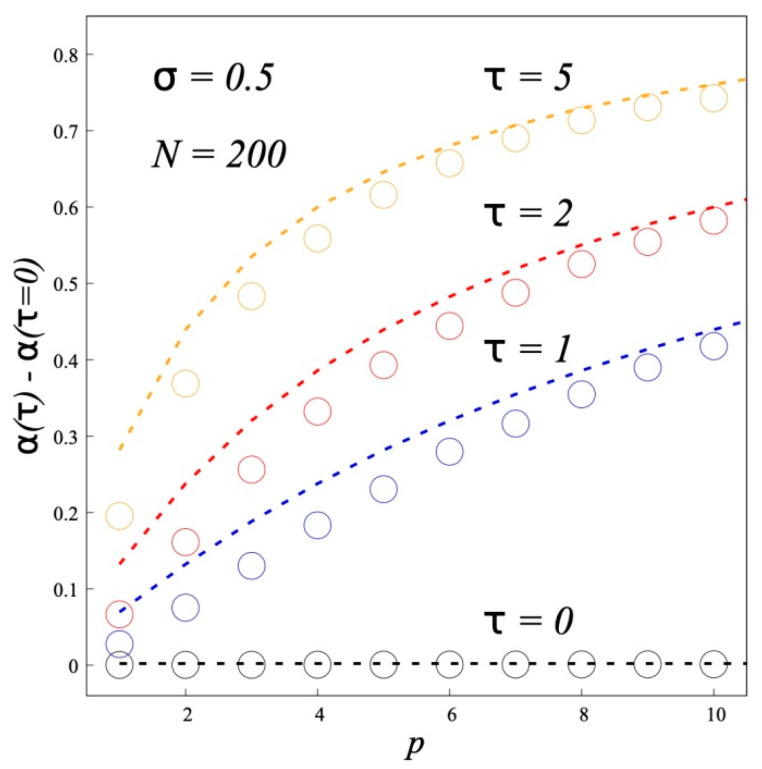
The fraction of “hairpins” α as a function of the statistical segment length *p* of the grafted chains. Dotted lines indicate theoretical curves, symbols indicate SF-SCF calculation results (*σ* = 0.5, *N* = 200).

Figure 8 compares results of the SF-SCF calculations of the fraction of “hairpins” *α* with the theoretical predictions. The values of *α* were calculated from the end segment distributions *g*(*z*). Note that the theory gives *α* > 0 even when *τ* = 0. This is an artifact of the use of a discrete two-state model to describe a continuous function *g*(*z*). To see the effect of the dipole-dipole interactions, we plot in Figure 8 the difference between the calculated *α*(*τ*) and *α*(*τ* = 0). One can see a surprisingly good agreement with the theory.

### 3.2. System of Two Interacting Brushes

In this section we consider the system of two opposing similar planar brushes located at a distance *D* from each other and immersed into the high-molecular solvent consisting of chains similar to the grafted ones. The section is divided into two subsections. In the first subsection we present results of calculation of the interaction free energy between brushes. In the second subsection we show how the brush structure changes when interacting with another brush.

#### 3.2.1. Free Energy of the Interaction between Two Brushes

**Figure 9:**
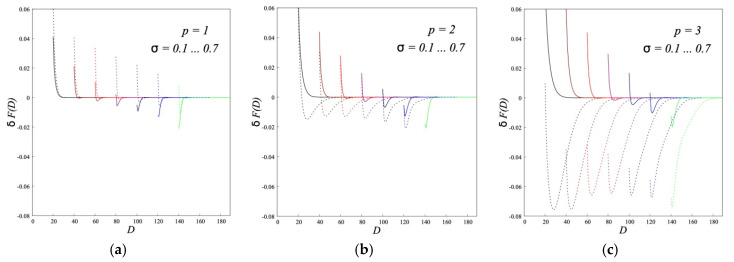
Interaction free energy *δF*(*D*) between two brushes at different grafting density of chains at *N* = 200 and various Kuhn length: *p* = 1 (**a**), *p* = 2 (**b**) and *p* = 3 (**c**). The grafting density varies from *σ* = 0.1 to *σ* = 0.7 in 0.1 increments, that is indicated by different colors from left to right. Solid lines in the graphs correspond to non-dipole brushes (*τ* = 0), dotted lines correspond to A-type brushes (*τ* = 5).

Free energy *δF*(*D*) (per unit of grafting area) of the interaction between two opposing brushes is defined as the difference between the free energy *F*(*D*) of the system at the given distance *D* and twofold energy of a single brush *F*(*D* = ∞). The free energy at a given value of *D* is calculated according to Equation (10). Results of the calculations of *δF*(*D*) for different σ at given *N* are shown in Figure 9. Data are presented for brushes from both non-dipole and dipolar chains with varying flexibility. The goal is to determine the effect of the limited flexibility of chains (*p* > 1) and the dipole-dipole interactions of longitudinal dipoles in A-type brushes (*τ* > 0).

Figure 9 shows that for non-polar flexible chains *p* = 1 at small σ the brushes repel each other. At larger *σ*, a small attraction is observed. The depth of the minimum increases with an increase of *σ*. Such a minimum was observed earlier in [11]. This attraction is an entropic effect. When entering the brush, the free chains from the melt lose more entropy than the chains of another brush. Therefore, the substitution of free chains by the grafted ones of another brush gives a gain of entropy. By an increase in the Kuhn length *p*, this gain decreases and the attraction between uncharged brushes shifts towards larger sigma (at *p* = 2) and disappears (at *p* = 3).

The presence of dipoles in the flexible chains *p* = 1 change the dependence *δF*(*D*) on *σ*. For all values of *σ* the brushes repel each other. For *p* > 1 one observes an opposite effect. At the same *τ* and *N*, an increase of *p* leads to a strong increase of the attraction between brushes (the depth of the negative minimum grows). The effect of σ is opposite to that for non-polar chains.

**Figure 10:**
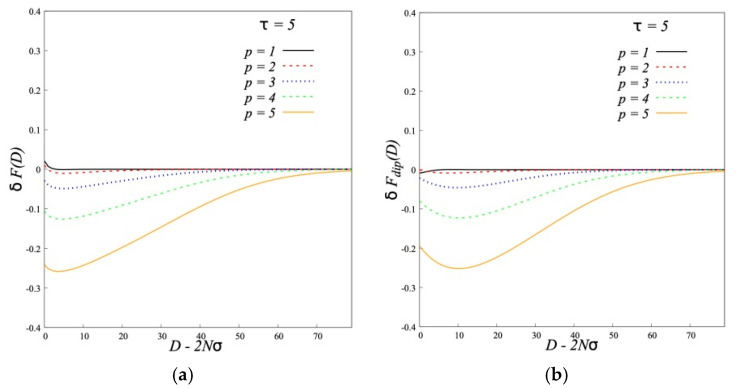
Interaction free energy *δF*(*D*) of two dipolar brushes (*τ* = 5) grafted to two parallel impenetrable surfaces (**a**). The dipole-dipole interactions *δF_dip_*(*D*) (**b**). In all cases, the degree of polymerization of the grafted chains and solvent molecules is equal to *N* = 200, the grafting density is *σ* = 0.3.

The analysis of data shows that such a behavior of *δF*(*D*) for dipolar brushes is determined by that of the dipolar contribution *δF_dip_*(*D*), where *Fdip* = *F_d−d_* −∑*_z,d_ u_d−d_*(*z*,*d*) *ϕ*(*z*,*d*) is the energy of dipole-dipole interactions in potential field *u_d−d_*(*z*,*d*) minus the work of the dipoles in this field. This is seen from the comparison of *δF*(*D*) and *δF_dip_*(*D*) (Figure 10).

With an increase in p the values of *δF_dip_* become more negative as the brushes approach each other indicating an attraction between them and go through the minimum. The decrease of *δF_dip_* begins at larger *D* and the depth of the minimum increases.

#### 3.2.2. Changes of Brush Structures by Their Interaction

**Figure 11:**
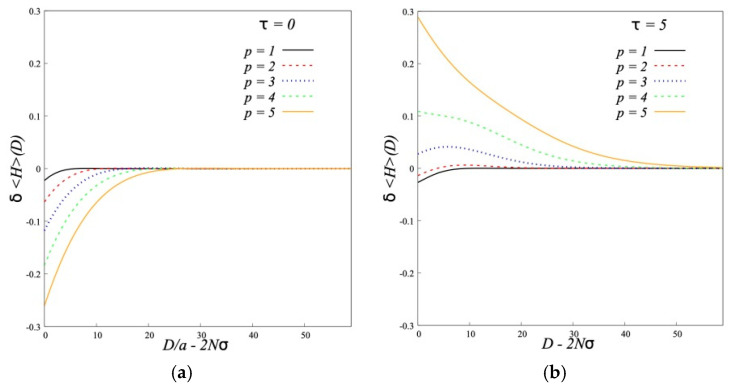
Change in first moment of polymer density distribution (*N* = 200, *σ* = 0.3) at *τ* = 0 (**a**) and *τ* = 5 (**b**).

**Figure 12:**
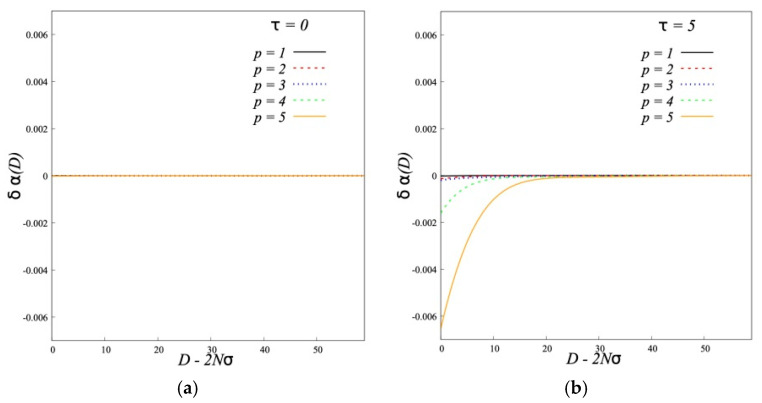
Change in fraction of backfolded chains (*N* = 200, *σ* = 0.3) at *τ* = 0 (**a**) and *τ* = 5 (**b**).

The effect of the interaction on the structure of individual brushes can be characterized by the changes of the brush thickness *δ*<*H*> and the fraction of hairpins *δα* (Figure 11 and Figure 12).

Figure 11 shows that for conventional brushes *τ* = 0 the grafted chains compress slightly by decrease of *D*. For dipolar brushes we see an opposite effect: the grafted chains slightly stretch. This stretching is energetically profitable because it leads to larger overlapping of chains belonging to different brushes with the alternative directions of dipole moments. The decrease of fraction of “hairpins” (Figure 12) shows that some backfolded chains unfold and penetrate into the opposite brush.

**Figure 13:**
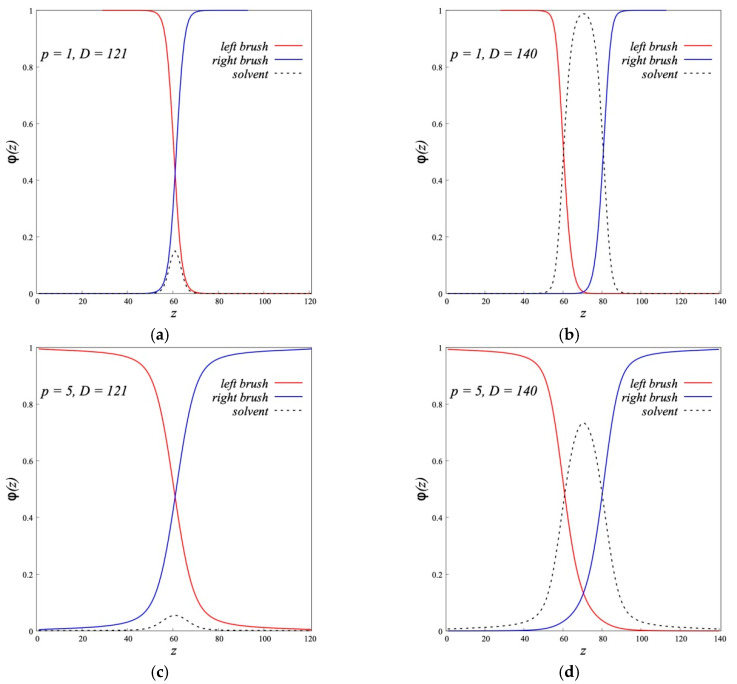
The volume fraction profiles of interacting dipolar (*τ* = 5) polymer brushes consisting of flexible (*p* = 1, *D* = 121 (**a**) and *D* = 140 (**b**)) and rigid (*p* = 5, *D* = 121 (**c**) and *D* = 140 (**d**)) polymer chains (*N* = 200, *σ* = 0.3).

The mutual penetration of the dipolar brushes is shown in Figure 13, that shows the density profiles of two opposing brushes at different distances *D* at *τ* = 5 for grafted flexible *p* = 1 and semi-flexible *p* = 5 chains. It is seen that the brushes overlap, and this overlapping is larger for more rigid chains. As can be seen from Figure 13, at *p* = 5 and *τ* = 5 at the approach of the brushes close to the limit, *D* = 121 = *Nσ* + 1, the chains of each of the opposing brushes pass through the entire counter brush, reaching its grafting surface. The polymer solvent is “squeezed” from the space between brushes by their compression.

**Figure 14:**
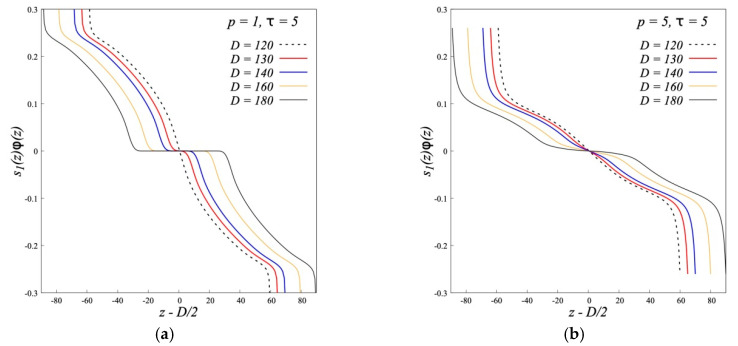
The segment orientation profile *s*_1_(*z*)*ϕ*(*z*) = *ϕ*(*z*, +1) − *ϕ*(*z*, −1) of two opposing dipolar brushes in the case of flexible (*p* = 1) (**a**) and rigid (*p* = 5) (**b**) polymer chains (*N* = 200, *σ* = 0.3).

The total parameter of the segments orientation of two opposing brushes is shown in Figure 14. For flexible chains (*p* = 1), with a decrease in *D* the dependencies for “left” and “right” brushes move to each other but practically do not change their shape. The plateau on the graphs for the brush polymers in Figure 14 remains when the brushes approach each other up to the smallest possible distance *D* = 2*aNσ*. For semi-flexible brushes *p* = 5, the plateau disappears already with a weak overlap of the peripheral layers of the opposing brushes and the curves for different *D* merge into one.

As we have shown in the first subsection the main contribution into the energy of interaction between two dipolar brushes in the similar solvent is provided by the dipole-dipole interactions. They lead to the attraction between brushes which increases with *p*. Analysis of the structural changes given in the second subsection allows to explain the increased attraction between dipolar brushes formed by semi-rigid chains.

## 4. Conclusions

By using the numerical lattice SF-SCF method, we have studied the effect of the increase in the Kuhn length of grafted chains on the structure and mutual interaction of two opposing planar non-polar and A-type dipolar brushes. Brushes are immersed in the solvent consisting of chains similar to the grafted ones. The degrees of polymerization as well as the parameters of flexibility and dipole-dipole interactions are the same for free chains in solution and chains in a brush.

It is shown that for brushes made of semi-rigid chains, as for brushes made of flexible chains, the modes of wet and dry brush are possible at low and high grafting densities, respectively. Reducing the chain flexibility leads to the loosening of the brush and to the increase of its thickness in the case of wet brush and to more gentle decline in brush density at the brush periphery, which contains free polymer, in the case of “dry” brush.

For unusual structure of A-type dipolar brushes the increase of the chain rigidity enhances the separation of grafted chains in a brush into two populations: backfolded chains with terminal monomers near the grafting surface and chains with the ends at the brush periphery. The fraction of backfolded chains grows by increase of the Kuhn segment length.

As to the interaction of opposite A-type dipolar brushes our main result is that the brushes from semi-rigid chains are attracted to each other at short distances. The attraction becomes more pronounced and begins at larger distances for more rigid chains at the same brush characteristics (polymerization degree, grafting density and dipole moments of monomer units). This effect of the chain rigidity is opposite to that for conventional brushes without dipoles in the chains. For such brushes the growth of the chain rigidity leads to the enhanced repulsion between them.

It is shown that the strong attraction of A-type dipolar brushes is energetically favorable and is connected with the dipole-dipole interactions. Chains of opposing brushes with oppositely directed dipoles penetrate deeply into each other upon contact. The decrease of fraction of «hairpins» shows that some backfolded chains unfold and penetrate into the opposite brush.

Note that the deep interpenetration of opposing brushes predicted in this work for unusual brushes was previously discussed for brushes with mesogenic groups in the backbone of semi-rigid grafted chains, undergoing a phase transition to a liquid-crystalline (LC) state [30,34]. The brushes were placed in a low molecular weight solvent and the transition was initiated by an increase in the orientation-dependent interaction of mesogenic groups. This transition occurs through the micro (nano)phase separated state of a brush (vertical phase segregation).

In this state, the grafted chains are divided into two populations [35]: a set of folded chains that form a dense internal LC microphase, and a set of more extended chains that form a microphase containing a solvent at the periphery of the brush. Upon contact of the brushes, the chains stretch, penetrating into the counter brush and forming a single LC phase. Brushes attract each other and even stick together. This is somewhat similar to what happens when A-type bipolar brushes made of semi-rigid chains approach each other, although the common property of the considered systems is only orientation-dependent interaction in the brushes.

All obtained results should be taken into account using the grafting of polymer chains on the surface of nanoparticles to prevent their aggregation in the polymer matrix. It is possible to obtain repulsive brushes from the A-type macromolecules by the alternative grafting of them at different ends. In this case the longitudinal dipole moments of any pair of differently grafted chains are directed antiparallel. As it was shown [18] by SF-SCF method, and confirmed for OLA brush by fully atomistic MD simulations the structure of such a brush is similar to the structure of conventional brushes without dipoles. As shown in this paper, conventional brushes from semi-rigid chains repel each other. As it was shown in [10], repulsive brushes can be obtained also in the case of chemical non-identity of the grafted chains in the brush and polymer macromolecules under the additional condition of attraction between them (the Flory interaction parameter χ < 0).

## Figures and Tables

**Table 1:** Scaling power-law dependencies of the brush thickness *H* and the penetration length Δ for different regimes of solvent penetration into the brush.

Regime	H	Δ	Δ/H
I	$N{\left(\frac{p\sigma }{M}\right)}^{1/3}$	$N{\left(\frac{p\sigma }{M}\right)}^{1/3}$	1
II	Nσ	$\frac{Np}{M\sigma }$	$M^{-1}\sigma^{-2}p$
III	Nσ	${\left(\frac{Np}{\sigma }\right)}^{1/3}$	$N^{-2/3}\sigma^{-4/3}p^{1/3}$
IV	${\left(Np\right)}^{\frac{1}{2}}$	${\left(Np\right)}^{\frac{1}{2}}$	1

## Appendix A. Derivation of the Diagram of States for Brushes in the Melt

### Appendix A.1. Regime I. Full Penetration

In regime I, the length Δ of the penetration zone between free chains and the brush chains is equal to the brush thickness:

(A1) $$\Delta_{\left(I\right)}=H_{\left(I\right)}$$

Free energy per one grafted chain in the mean field approximation is

(A2) $$F\sim \frac{H^{2}}{Np}+\frac{\varphi^{2}}{M}\cdot \frac{H}{\sigma }\sim \frac{H^{2}}{Np}+\frac{N^{2}\sigma }{HM},$$

where the first term is the contribution of the elastic stretching of the grafted chains, and the second term corresponds to the entropy of mixing of the brush chains and free solvent chains.

Minimization of free energy with respect of the brush thickness gives dependence:

(A3) $$H_{\left(I\right)}\sim N{\left(\frac{\sigma p}{M}\right)}^{1/3}$$

The mean volume fraction of grafted polymer is equal to

(A4) $$\varphi =\frac{N\sigma }{H}={\left(\frac{M\sigma^{2}}{p}\right)}^{1/3}$$

Since *ϕ* < 1 corresponds to full overlapping of the polymer and solvent, the upper limit of regime I in the diagram is described by the equation:

(A5) $$\sigma_{\left(I-II\right)}={\left(\frac{p}{M}\right)}^{1/2}$$

### Appendix A.2. Regime II. Partial Penetration

In the partial penetration regime, the average density of monomeric units of the grafted chains becomes close to the maximum *ϕ* ≈ 1, therefore, the brush thickness is described by the scaling dependence

(A6) $$H_{\left(II\right)}\sim N\sigma$$

The width of the penetration zone Δ is determined from the minimum energy for free chains:

(A7) $$F\sim \frac{\Delta^{2}}{Np}+\frac{\Delta }{M\sigma },\;\;\frac{dF}{d\Delta }=0\Rightarrow \Delta_{\left(II\right)}=\frac{Np}{M\sigma }$$

The given scaling dependence for free energy in the partial penetration regime is a generalization of the conclusions from [9,36].

### Appendix A.3. Regime III. Peripheral Penetration

When the partial penetration becomes very small, peripheral penetration is occurred. In this regime the brush is collapsed

(A8) $$H_{\left(III\right)}\sim N\sigma$$

The mobile chains are expelled from the brush volume except for the peripheral region, where the volume fraction of the brush-forming chains sharply decreases from *ϕ*(*z*) = 1 in the interior of the brush down to *ϕ*(*H*) = 0.

Penetration length can be obtained by minimizing free energy [9,36]:

(A9) $$F\sim \frac{\Delta^{2}}{Np}+\frac{1}{\sigma \Delta },\;\;\frac{dF}{d\Delta }=0\Rightarrow \Delta_{\left(III\right)}={\left(\frac{Np}{\sigma }\right)}^{1/3}$$

The boundary between regimes II and III is determined from equality Δ_(*II*)_ = Δ_(*III*)_, that gives the expression

(A10) $$\sigma_{\left(II-III\right)}=\frac{Np}{M^{3/2}}$$

### Appendix A.4. Regime IV. Unstretched Gaussian Chains

This regime corresponds to the region of sufficiently low grafting densities, at which the grafted chains are not stretched and have an unperturbed Gaussian size:

(A11) $$H_{\left(IV\right)}\sim \sqrt{Np}$$

The solvent molecules freely penetrate into the brush, so the value of *H*_(*IV*)_ is equal to Δ_(*IV*)_.

The boundaries between regimes I and IV, as well as between regimes III and IV are determined from the equalities *H*_(*I*)_ = *H*_(*IV*)_ and *H*_(*III*)_ = *H*_(*IV*)_, respectively, as

(A12) $$\sigma_{\left(I-IV\right)}=\frac{p^{1/2}M}{N^{3/2}}$$

and

(A13) $$\sigma_{\left(III-IV\right)}={\left(\frac{p}{N}\right)}^{1/2}$$

## References

[1] Alexander S. (1977). Adsorption of chain molecules with a polar head a scaling description. J. Phys..

[2] De Gennes P. (1980). Conformations of Polymers Attached to an Interface. Macromolecules.

[3] Kumar S., Jouault N., Benicewicz B., Neely T. (2013). Nanocomposites with polymer grafted nanoparticles. Macromolecules.

[4] Chen W.L., Cordero R., Tran H., Ober C. (2017). 50th anniversary perspective: Polymer brushes: Novel surfaces for future materials. Macromolecules.

[5] Kreer T. (2016). Polymer-Brush Lubrication: A Review of Recent Theoretical Advances. Soft Matter.

[6] Brinson L., Deagen M., Chen W., McCusker J., McGuinness D., Schadler L., Palmeri M., Ghumman U., Lin A., Hu B. (2020). Polymer Nanocomposite Data: Curation, Frameworks, Access, and Potential for Discovery and Design. ACS Macro Lett..

[7] Birshtein T., Amoskov V. (2000). Polymer brushes. Polym. Sci. C.

[8] Zhulina E., Borisov O., Brombacher L. (1991). Theory of a planar grafted chain layer immersed in a solution of mobile polymer. Macromolecules.

[9] Borukhov I., Leibler L. (2002). Enthalpic Stabilization of Brush-Coated Particles in a Polymer Melt. Macromolecules.

[10] Witten T., Leibler L., Pincus P. (1990). Stress relaxation in the lamellar copolymer mesophase. Macromolecules.

[11] Wijmans C., Zhulina E., Fleer G.J. (1994). Effect of Free Polymer on the Structure of a Polymer Brush and Interaction between Two Polymer Brushes. Macromolecules.

[12] Ginzburg V. (2019). Recent Developments in Theory and Modeling of Polymer-Based Nanocomposites. Probl. Nonlinear Mech. Phys. Mater..

[13] Gay C. (1997). Wetting of a Polymer Brush by a Chemically Identical Polymer Melt. Macromolecules.

[14] Balazs A., Singh C., Zhulina E. (1998). Modeling the Interactions between Polymers and Clay Surfaces through Self-Consistent Field Theory. Macromolecules.

[15] Glova A., Falkovich S., Larin S., Mezhenskaia D., Lukasheva N., Nazarychev V., Tolmachev D., Mercurieva A., Kenny J., Lyulin S. (2016). Poly(lactic acid)-based nanocomposites filled with cellulose nanocrystals with modified surface: All-atom molecular dynamics simulations. Polym. Int..

[16] Glova A., Larin S., Falkovich S., Nazarychev V., Tolmachev D., Lukasheva N., Lyulin S. (2017). Molecular dynamics simulations of oligoester brushes: The origin of unusual conformations. Soft Matter.

[17] Stockmayer W. (1967). Dielectric dispersion in solutions of flexible polymers. Pure Appl. Chem..

[18] Birshtein T., Polotsky A., Glova A., Amoskov V., Mercurieva A., Nazarychev V., Lyulin S. (2018). How to fold back grafted chains in dipolar brushes. Polymer.

[19] Birshtein T.M., Ptitsyn O.B. (1966). Conformations of Macromolecules (High Polymers).

[20] Kumar R., Sumpter B., Muthukumar M. (2014). Enhanced Phase Segregation Induced by Dipolar Interactions in Polymer Blends. Macromolecules.

[21] Mahalik J., Sumpter B., Kumar R. (2016). Vertical phase segregation induced by dipolar interactions in planar polymer brushes. Macromolecules.

[22] Budkov Y., Kalikin N., Kolesnikov A. (2017). Polymer chain collapse induced by many-body dipole correlation. Eur. Phys. J. E.

[23] Gordievskaya Y., Kramarenko E., Budkov Y. (2018). An interplay of electrostatic and excluded volume interactions in the conformational behavior of a dipolar chain: Theory and computer simulations. Soft Matter.

[24] Mahalik J., Sumpter B., Kumar R. (2017). Attraction between Opposing Planar Dipolar Polymer Brushes. Langmur.

[25] Glova A., Larin S., Nazarychev V., Karttunen M., Lyulin S. (2020). Grafted Dipolar Chains: Dipoles and Restricted Freedom Lead to Unexpected Hairpins. Macromolecules.

[26] Scheutjens J., Fleer G. (1979). Statistical theory of the adsorption of interacting chain molecules. 1. Partition 458 function, segment density distribution, and adsorption isotherms. J. Phys. Chem..

[27] Fleer G.J., Cohen Stuart M.A., Scheutjens J.M.H.M., Cosgrove T., Vincent B. (1993). Polymers at Interfaces.

[28] Kuhn W., Kuhn H. (1948). Rigidity of chain molecules and its determination from viscosity and flow birefringence in dilute solutions. J. Colloid Sci..

[29] Van der Linden C., Leermakers F., Fleer G. (1995). Adsorption of Semiflexible Polymers. Macromolecules.

[30] Amoskov V., Birshtein T., Pryamitsyn V. (1996). Theory of Polymer Brushes of Liquid-Crystalline Polymers. Macromolecules.

[31] Leermakers F., Scheutjens J., Gaylord R. (1984). Modelling the amorphous phase of a melt crystallized, semicrystalline polymer: Segment distribution, chain stiffness, and deformation. Polymer.

[32] Wijmans C., Leermakers F., Fleer G. (1994). Chain stiffness and bond correlations in polymer brushes. J. Chem. Phys..

[33] Eslami H., Rahimi M., Müller-Plathe F. (2013). Molecular Dynamics Simulation of a Silica Nanoparticle in Oligomeric Poly(methyl methacrylate): A Model System for Studying the Interphase Thickness in a Polymer–Nanocomposite via Different Properties. Macromolecules.

[34] Amoskov V., Birshtein T., Pryamitsyn V. (1998). Theory of LC polymer brushes: Interpenetrating brushes. Macromolecules.

[35] Klushin L., Birshtein T., Amoskov V. (2001). Microphase coexistence in brushes. Macromolecules.

[36] Semenov A. (1985). Contribution to the theory of microphase layering in block-copolymer melts. JETP.

